# Acute Copper Toxicity: Succimer Makes you Less Blue

**DOI:** 10.1007/s13181-026-01117-9

**Published:** 2026-03-02

**Authors:** Ivan Ivanov, Waleed Abouelela, Tyler Debbie, Denise Fernández, Lewis S. Nelson, Anita Mudan

**Affiliations:** 1 Department of Emergency Medicine, Rutgers New Jersey Medical School, Newark, NJ USA; 2 New Jersey Poison Control Center, Newark, NJ USA; 3 Robert Wood Johnson New Brunswick Department of Emergency Medicine, New Brunswick, NJ USA; 4 Florida Atlantic University Schmidt College of Medicine, Boca Raton, FL USA

**Keywords:** Copper, Hemolytic anemia, Caustic, Fungicide, Succimer

## Abstract

**Introduction:**

Acute copper toxicity is a rare occurrence, and management guidelines are not based on robust evidence. Treatment recommendations are often extrapolated from those of chronic copper intoxication and the management of Wilson’s disease. Although D-penicillamine, EDTA, and dimercaprol have varying success, we describe the use of chelation with succimer monotherapy in a patient with acute copper salt ingestion with subsequent improvement of clinical status and copper concentrations.

**Case Report:**

A 38-year-old woman presented to an ED after ingestion of liquid copper fungicide containing 27.5% copper diammonia diacetate complex. The regional poison center initially recommended D-penicillamine along with supportive care. Due to the lack of availability of D-penicillamine, succimer was chosen as an alternative agent. The patient developed mild hemolysis and liver injury during her stay, but did not develop any kidney injury. She had a Zargar 2A caustic injury to the stomach and duodenal bulb. She received succimer for a total of 10 days, and copper concentrations decreased from 1,295 mcg/dL (reference range: 80–158 mcg/dL) to normal levels before discharge.

**Discussion:**

There is limited evidence surrounding chelation therapy in acute copper poisoning. We describe a case of a patient who clinically improved while receiving aggressive supportive care and associated succimer monotherapy after acute ingestion of a copper compound.

## Introduction

Copper is an essential element and is the third most abundant trace element in the body [1]. It is utilized by the body for its redox activity, and its homeostasis is meticulously managed. Acute copper poisoning, although rare, can lead to significant morbidity, with mortality rates ranging from 14–36% [2]. Copper induces oxidative stress through the Fenton and Haber-Weiss reactions, as well as directly through glutathione depletion [3]. Acute ingestion of copper salts primarily results in hemolysis, acute liver and kidney injury, and corrosive injury to the gastrointestinal tract [4, 5]. 

Management of acute copper poisoning involves decontamination when indicated, supportive care, and chelation therapy [2]. Chelation in patients with acute copper toxicity is extrapolated from the management of those with chronic copper overload syndromes, such as Wilson’s disease, and D-penicillamine is often recommended as the first-line chelating agent [6]. Other chelators such as EDTA and dimercaprol have also been suggested [2]. We describe a case of an acute copper fungicide ingestion containing copper diammonia diacetate, an organocopper compound rarely documented in ingestion, which was chelated with oral succimer and ultimately recovered.

## Case Summary

**Fig. 1:**
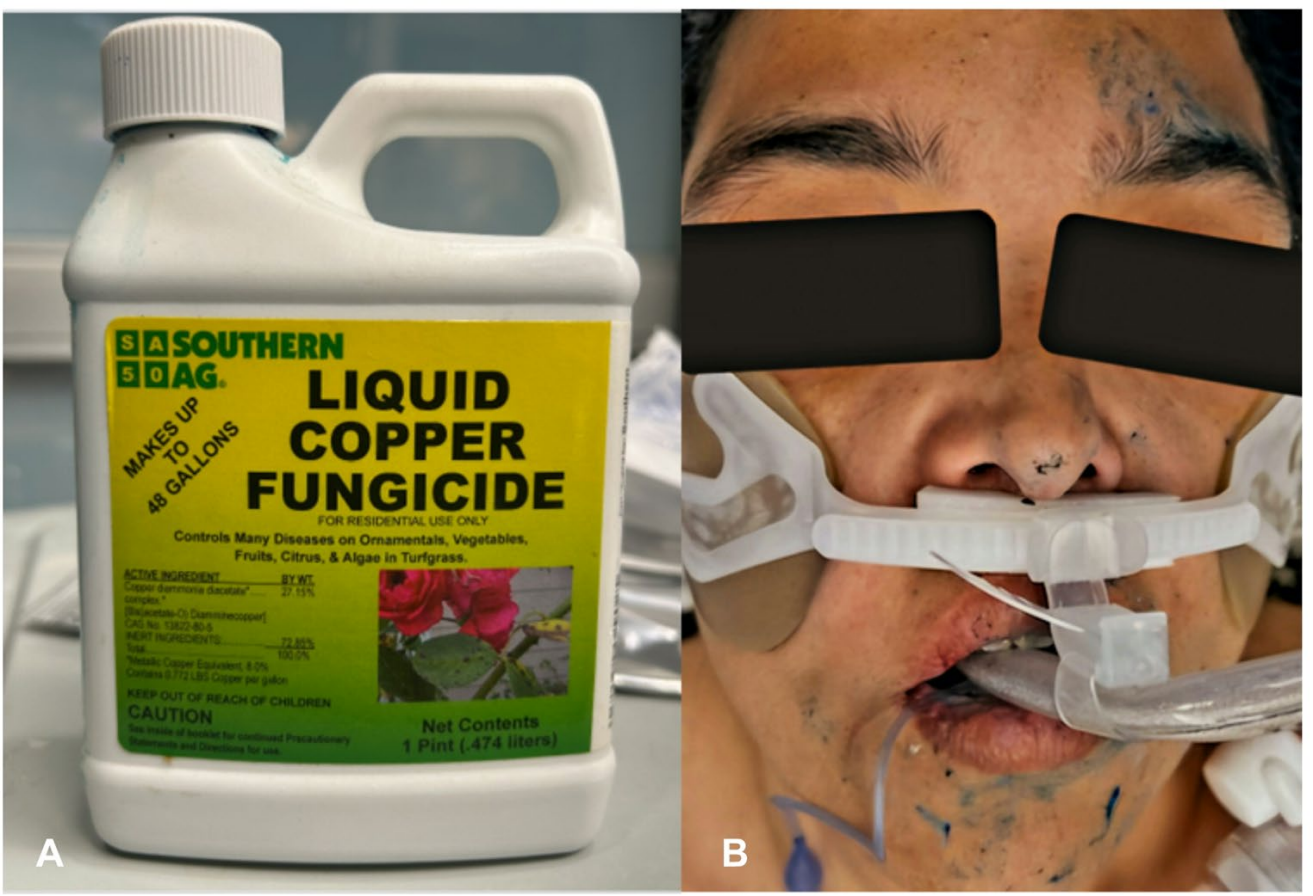
**(A**) Image of copper fungicide product showing 27.15% copper diammonia diacetate and 0.772 pounds of copper per gallon. (**B**) Blue residue around the patient’s face after intubation

A 38-year-old woman presented to the Emergency Department (ED) after ingesting approximately 250 ml of “Southern Ag” brand liquid copper fungicide containing 27.15% copper diammonia diacetate, 8% elemental copper by weight, in a suicide attempt (Fig. 1A). Emergency medical services were called after the patient’s husband found her on a riverbank next to a bottle containing a bright blue liquid. She was intubated for airway protection secondary to depressed mental status and vomiting. Upon presentation to the ED, she had blue residue in her oropharynx, face, and hands (Fig. 1B). Initial vital signs were blood pressure 131/96 mmHg, heart rate 140 beats per minute, temperature 36.1° Celsius, and 100% oxygen saturation on the ventilator. The patient’s skin was decontaminated with soap and water, and an orogastric tube was placed with return of 70 mL of bright blue fluid. There was 92.5 g per liter of this product; therefore, with 180 mL ingested post-decontamination assuming no dilution, she likely ingested approximately 16.7 g of elemental copper. She was sedated with propofol and fentanyl, and she subsequently developed hypotension requiring a crystalloid fluid bolus and infusion of norepinephrine. Shortly thereafter, the patient had large-volume bloody diarrhea with blue color and was given one unit of packed red blood cells due to concern for acute gastrointestinal hemorrhage contributing to her hypotension.

The poison center was contacted and recommended chelation with D-penicillamine. However, due to a lack of availability in the area, succimer was initiated at 10 mg/kg three times daily for five days, followed by twice daily for fourteen days based on dosing for lead toxicity. Additionally, N-acetylcysteine (NAC) was started for hepato-protection and its potential chelating ability. Initial labs were notable for white blood cell (WBC) count 9.80 × 10^3^/µL, hemoglobin 14.7 g/dL, sodium 137 mEq/L, potassium 4.5 mEq/L, blood urea nitrogen 11 mg/dL, creatinine 0.8 mg/dL, aspartate aminotransferase 29 U/L, alanine aminotransferase < 6 U/L, alkaline phosphatase 55 U/L, lactate 2.0 mmol/L, INR 1.11, negative acetaminophen concentration, negative salicylate concentration, negative ethanol concentration and a methemoglobin of 1.1%. Computed tomography of the chest, abdomen, and pelvis showed diffuse gastroenteritis and colitis without radiopaque material. The patient was admitted to the intensive care unit.

On hospital day 1 (HD1), the patient underwent esophagogastroduodenoscopy, showing Zargar 2A ulcerations to the stomach and duodenal bulb. Her hemoglobin decreased to 9.3 g/dL, for which she was given another unit of packed red blood cells. The patient’s lactate increased from 2.0 mmol/L to greater than 17 mmol/L approximately six hours after presentation, and decreased to 2.9 mmol/L within 90 min following approximately one liter of intravenous fluids. Furthermore, she developed a leukocytosis to 24.20 × 10^3^/µL, and a transaminitis with indirect hyperbilirubinemia approximately eight hours after presentation. She was continued on succimer, NAC, and maintenance IV crystalloid. On HD3, she developed jaundice and scleral icterus with repeat laboratory analysis showing an undetectable haptoglobin (< 8.1 mg/dL), total bilirubin 4.3 mg/dL with an indirect bilirubin of 3.7 mg/dL, consistent with hemolysis. Other laboratory values at that time were AST 83 IU/L, ALT 15 IU/L, albumin 2.9 g/dL, and INR 1.13, as shown in Table 1.

**Table 1:** Lab concentration trends throughout hospitalization

Lab	0	1	2	3	4	5	6	7	8	9
Cu (ug/dL)	1295	111	84	90	108	109	107			
Hgb (g/dL)	14.7	11	9.8		9.3			9.7	8.4	8
LDH (U/L)		379	877	806	589	606	484	499	357	
Lactic acid (mmol/L)	1.3–2.0	2.9->17	1.5	0.7						
Haptoglobin (mg/dL)		13.1	< 8.1	< 8.1	18	26	44	55		
AST (U/L)	29	42	90		99		68			54
ALT (U/L)	6	12	16		52		66			28
TBILI (mg/dL)	0.6	2.5	4.8		2.2		1.2			1
Cr (mg/dL)	0.8	1.1	0.9		0.6		0.6			1
Cu Ur Spot (ug/g)					295					
Cu 24 h (ug/L)				121						

**Fig. 2:**
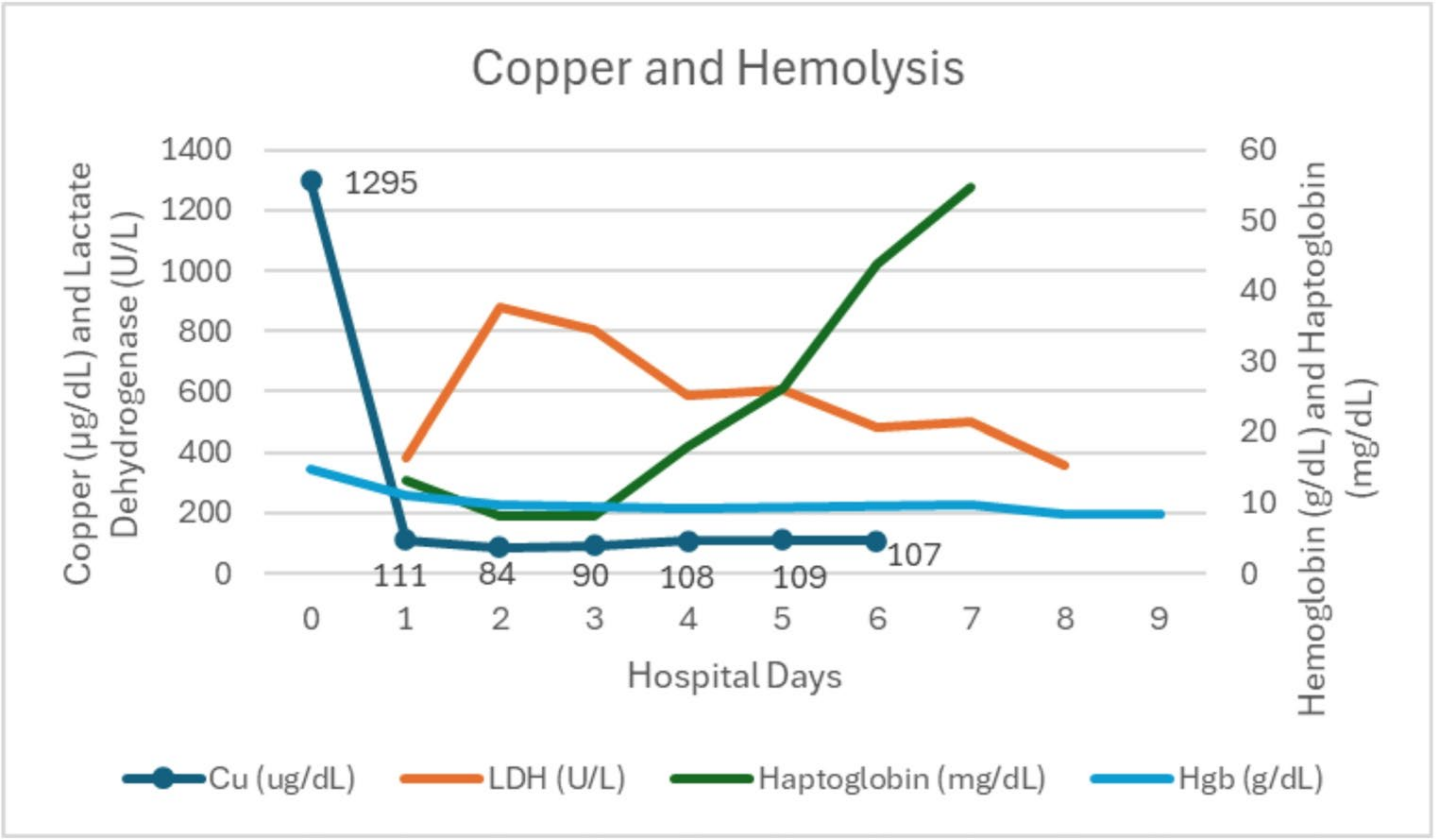
Graphical depiction of copper, hemoglobin, haptoglobin, and LDH (to depict hemolysis) values throughout hospitalization

Over the next two days, the patient’s jaundice and scleral icterus improved. On hospital day 5, copper concentrations from hospital days 0–2 returned, revealing an initial serum copper concentration of 1,295 mcg/dL (reference range 80–158 mcg/dL) with subsequent hospital days 2 and 3 noting concentrations of 111 and 84 mcg/dL, respectively (Fig. 2). The patient developed a mild ALT increase to 67 IU/L, raising concern for developing liver injury, but her synthetic function remained normal and markers of hemolysis improved. Given her gastric injury, she remained nil per os (NPO) and was initiated on total parenteral nutrition (TPN) with supplemental zinc to induce metallothionein synthesis in the intestine and inhibit absorption of copper. Her presenting zinc concentration returned later at 38 mcg/dL (reference range 44–115 mcg/dL).

The patient’s clinical status continued to improve over the following week. Random urine copper concentrations from hospital day 4 (first day of sampling) were elevated at 295 mcg/g creatinine, peaking on day 5 (352 mcg/g) followed by a decline throughout the remainder of her hospital course. These were obtained to assess elimination as no clinical laboratories contacted would assess fecal copper concentrations. The patient was discharged after a 13-day hospitalization with prescriptions for the remainder of the succimer course, as well as continued zinc supplementation. *Consent for publication of this case was obtained and provided to the journal in accordance with JMT policy*.

## Discussion

We describe a case of acute copper poisoning following ingestion of copper diammonia diacetate. The patient developed hemolysis and gastrointestinal caustic injury; however, she never developed significant liver or kidney injury, commonly seen in acute copper salt ingestions. Post exposure, copper is taken up into hepatocytes, creating reactive oxygen species and inciting oxidative stress, leading to direct liver injury and centrilobular necrosis [6]. NAC was administered for this patient for general hepatoprotection and possible chelating ability. It was discontinued on HD3 due to stable liver function test results. Hemolysis secondary to acute copper ingestion occurs both early and late post-ingestion via two different mechanisms [7]. The early hemolysis occurs within 12–24 h of ingestion and is due to a reaction with the sulfhydryl-rich erythrocyte cell membrane, leading to membrane lipid peroxidation, resulting in increased permeability and rigidity of the erythrocyte membrane [7]. The late mechanism of hemolysis occurs after hepatocyte necrosis and the re-release of copper into the bloodstream [4]. Copper can also oxidize the iron in hemoglobin and can lead to methemoglobinemia [4]. Nephrotoxicity from copper is caused by direct oxidative stress to the proximal tubule that can cause acute tubular necrosis, which can also be secondary to hemoglobinuria [4]. Copper salts are directly corrosive to the gastrointestinal endothelium through the production of reactive oxygen species, and can potentially alter barrier functions, increasing passive paracellular transport and decreasing efflux proteins, like p-glycoprotein [5, 8]. In acute ingestion and in vitro studies, caustic injury further increases the absorption of copper and other xenobiotics [8]. Our patient also developed a hyperlactatemia that preceded the onset of hemolysis and was likely secondary to the hypotension, caustic injury, and cellular death in the GI tract. A case report of a patient with a similar copper ammonia complex ingestion who was found dead at home had a post-mortem plasma copper concentration of 500 ug/dL [9].

In addition to supportive measures, other acute treatments include decontamination and chelation. Activated charcoal has no data to support its efficacy following acute copper ingestion, and could limit the diagnostic utility of endoscopy. Gastric lavage has limited utility due to the vomiting induced by copper, a historical emetic [2]. Orogastric suction has been used in the initial management following ingestion to reduce the copper burden, although the value remains unclear.

Chelation is a challenge due to the lack of sufficient safety and efficacy data and availability of chelators historically used in copper poisoning. D-penicillamine is often recommended as the first-line chelator for acute copper toxicity, extrapolated from its effectiveness in Wilson’s disease. However, data on its use in acute copper toxicity are limited. Furthermore, there are even less data on chelation of organocopper compounds, with a prior case report attempting chelation with dimercaprol [10]. In vitro studies have shown relatively similar protection against copper sulfate-induced hemolysis when red blood cells are treated with D-penicillamine or succimer [11], which was the primary evidence to support the use of succimer in this case. Additionally, there are murine models showing increased elimination of copper when treated with succimer [12]. There is also a prior case report of a copper sulfate ingestion in which chelation with succimer was given and the patient had a good clinical outcome [13]. Succimer is also generally well-tolerated; however, there is a concern for increased urinary elimination of copper with succimer, potentially leading to nephrotoxicity [4]. D-penicillamine is less commonly available and usually found at tertiary centers that care for patients with Wilson’s disease. It also faces more drug shortages compared to succimer, which is more readily available [14]. Especially relevant to this patient’s management was the use of an oral chelator in the setting of gastrointestinal caustic injury. With the caustic injury, there was concern about the efficacy of the oral chelating agent; however, there was a significant reduction in the patient’s serum copper levels while succimer monotherapy was administered. Another note, is that NAC may act as a potential chelator of copper due to its thiol capability of binding, which can decrease copper uptake into cells; however, its interactions with copper-mediated reactive oxygen species have not been fully elucidated [15]. 

Limitations to our case report include the lack of causality between succimer administration and the absence of significant end-organ damage from this copper ingestion. Urinary elimination, typically a minor route of elimination for copper, was increased when compared to baseline urinary copper concentrations in unexposed subjects. However, this may have been due to the large initial serum concentration of copper following the ingestion. Additionally, the contribution of a dilutional effect from intravenous fluids on this patient’s decreasing copper concentration is unknown. Another limitation is that whole blood concentrations of copper were not obtained, and that serum concentrations might not accurately reflect the pharmacokinetics of the ingested copper. Moreover, urine copper concentrations were not collected pre- and post-succimer therapy, leading to an inability to compare elimination rates. Ingestion of this specific copper compound may have different absorption, metabolism, distribution, elimination or clinical manifestations compared to inorganic copper salts.

## Conclusion

We report a case of survival after organic copper liquid ingestion with evidence of significant systemic and gastrointestinal toxicity. The patient responded well to supportive care and use of succimer as a chelating agent. We believe this report may serve as a catalyst for implementing further studies evaluating the effectiveness of succimer as a mono-therapy chelator for acute copper toxicity.

## Declarations

Consent for publication of this case was obtained and provided to the journal in accordance with JMT policy.
